# Complete Genome Sequence of *Methylobacterium* sp. Strain AMS5, an Isolate from a Soybean Stem

**DOI:** 10.1128/genomeA.00144-16

**Published:** 2016-03-17

**Authors:** Tomoyuki Minami, Yoshiyuki Ohtsubo, Mizue Anda, Yuji Nagata, Masataka Tsuda, Hisayuki Mitsui, Masayuki Sugawara, Kiwamu Minamisawa

**Affiliations:** Graduate School of Life Sciences, Tohoku University, Aoba-ku, Sendai, Japan

## Abstract

Nonrhizobial *Methylobacterium* spp. inhabit the phyllosphere of a wide variety of plants. We report here the complete genome sequence of *Methylobacterium* sp. AMS5, which was isolated from a soybean stem. The information is useful for understanding the molecular mechanisms of the interaction between nonrhizobial *Methylobacterium* spp. and plants.

## GENOME ANNOUNCEMENT

*Methylobacterium* is a genus of facultative methylotrophic bacteria that are able to utilize C1 compounds, including methanol and methylamine (1). Some members of this genus inhabit the phyllosphere of various plant species, including *Arabidopsis thaliana*, rice, and soybean (2–4). It has also been reported that the community composition of nonrhizobial *Methylobacterium* in the phyllosphere was determined by the habits and plant species (2) and changed in response to the level of nitrogen fertilizer and nodulation phenotype in soybean (4). However, the genetic and metabolic systems involved in the interaction between *Methylobacterium* spp. and plants are poorly understood. In this study, we sequenced the genome of *Methylobacterium* sp. AMS5, which was previously isolated from the stem of a soybean (5), to explore the molecular mechanism for specific interactions between *Methylobacterium* and soybean plants.

The genome of strain AMS5 was sequenced using the 454 GS-FLX Titanium kit (Roche, Basel, Switzerland) and the Illumina HiSeq 2000 system (Illumina, San Diego, CA, USA). A fragmented genome library was constructed for 454 GS-FLX Titanium, and a 3-kb mate-pair genome library was constructed for Illumina HiSeq sequencing. The mate pairs were extracted and trimmed by ShortReadManager (6), in which 21-mers occurring more than six times were regarded as valid. The reads from these two systems were assembled by using Newbler version 2.6 (Roche). The finishing was facilitated by using GenoFinisher and AceFileViewer (6), in which 75 gaps were closed *in silico*, and seven gaps were closed by PCR with primers designed by GenoFinisher, and the reaction products were sequenced with the Sanger method by using a 3130xl DNA analyzer with a BigDye Terminator cycle sequencing reaction kit (Life Technologies Corporation, Carlsbad, CA, USA). We also conducted nine PCR experiments to solve repeat-induced ambiguities. The finished sequence was validated by FinishChecker (6). The AMS5 sequence was annotated using the NCBI Prokaryotic Genome Automatic Annotation Pipeline (7), and the result was manually inspected with respect to positions of start codons for predicted open reading frames using the Microbial Genome Annotation Pipeline (MiGAP; http://www.migap.org) and GenomeMatcher (8).

The genome of strain AMS5 consists of four replicons: a single chromosome (5,435,450 bp, 68.4% GC) and three plasmids designated as pAMS5a (117,697 bp, 65.3% GC), pAMS5b (25,608 bp, 65.3% GC), and pAMS5c (20,451 bp, 66.3 % GC). The genome structure of the chromosome was extremely similar to those of *M. extorquens* strains, whereas the three plasmids were unique to AMS5. The genes involved in tetrahydromethanopterin and tetrahydrofolate-dependent methanol utilization pathways and the *N*-methylglutamate pathway for methylamine utilization (*gmaS*, *mgsABC*, *mgdABCD*) were predicted, suggesting that strain AMS5 could utilize both methanol and methylamine as C1-carbon sources. It is the first report of a genome sequence of a nonrhizobial *Methylobacterium* sp. isolated from a leguminous plant. The genome information is useful for understanding the specific interactions between *Methylobacterium* spp. and soybeans.

### Nucleotide sequence accession numbers.

The genome sequence of *Methylobacterium* sp. AMS5 has been deposited at the DDBJ/EMBL/GenBank under the accession numbers CP006992 through CP006995.
